# Refractory acute generalized exanthematous pustulosis—Successfully treated with bimekizumab

**DOI:** 10.1016/j.jdcr.2026.03.023

**Published:** 2026-04-06

**Authors:** Flurin L. Brand, Susanne Radonjic-Hoesli, Christoph Schlapbach

**Affiliations:** Department of Dermatology, Inselspital, Bern University Hospital, Bern, Switzerland

**Keywords:** AGEP, bimekizumab, biologic, drug eruption, interleukin-17A/F inhibition

## Introduction

Acute generalized exanthematous pustulosis (AGEP) is a rare, usually drug-induced, severe cutaneous adverse effect. Standard therapy includes withdrawal of the causative agent and often systemic corticosteroids, yet refractory cases remain challenging.^1^

Bimekizumab is an interleukin (IL)-17A/F inhibitor approved for the treatment of psoriasis and psoriatic arthritis. To date, the efficacy of bimekizumab in AGEP and the contribution of IL-17F to AGEP pathogenesis remains unclear. In this case report, we describe the successful and rapid resolution of AGEP in a patient treated with bimekizumab.

## Case report

A 64-year-old woman with a history of guttate psoriasis presented with a rapidly progressive pruritic eruption after treatment for pneumonia. Prior to onset, she had received clarithromycin for 3 days, amoxicillin for 7 days, and inhaled budesonide/formoterol plus acetylcysteine.

**Fig 1:**
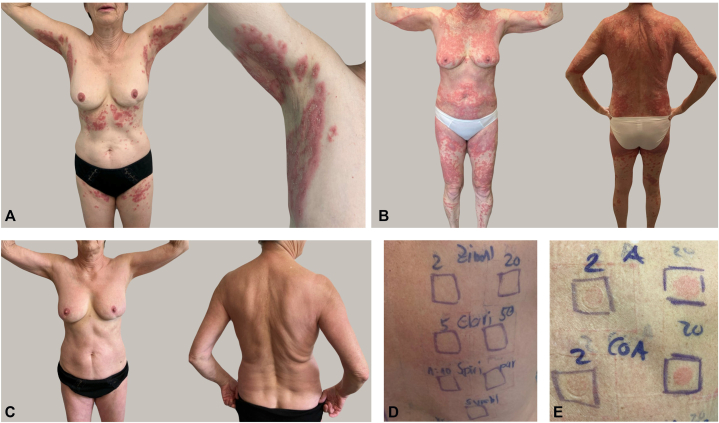
AGEP. **A,** At the initial presentation, erythematous plaques with numerous pinhead-sized pustules and fine lamellar scaling were observed in the armpits, submammary region and groin. **B,** Clinical picture after 2 weeks of systemic cortisone therapy. **C,** Clinical picture 1 week after the first injection with bimekizumab. **D** and **E,** Patch test revealed delayed-type hypersensitivity reactions to both amoxicillin and amoxicillin/clavulanic acid.

Approximately 9 days after antibiotic initiation, she developed an intensely pruritic eruption beginning in the axillae and spreading rapidly. Ten days later, at initial presentation to our clinic, she exhibited extensive erythematous plaques with numerous pinhead-sized pustules and fine scaling in the axillae, groin, and buttocks, without mucosal involvement (Fig 1, *A*). The patient received systemic corticosteroids for a total of 21 days (initial oral prednisolone followed by dexamethasone; see Table I for detailed dosing and timeline). Despite continuous systemic therapy, the eruption progressed clinically with increasing pustulation and persistent pruritus. No objective improvement was observed, and her symptoms significantly impaired her quality of life (Fig 1, *B*).

**Table I:** Clinical course and medication timeline

A lesional biopsy showed spongiform subcorneal and intraepidermal pustules, papillary dermal edema and a perivascular infiltrate with very sparse interspersed eosinophils. Given the temporal relationship, a drug-induced AGEP triggered by amoxicillin was suspected. All antibiotics had been discontinued 12 days prior to presentation at our clinic. Nevertheless, the eruption continued to worsen despite drug withdrawal and systemic corticosteroid therapy.

Because of disease progression and refractoriness to corticosteroids, off-label bimekizumab therapy (2 × 160 mg SC) was initiated after written informed consent, and systemic corticosteroids were discontinued at the time of bimekizumab initiation. Within 1 week, complete clearance of pustules and erythema was observed (Fig 1, *C*). The patient reported sustained remission and absence of pruritus during a follow-up period of 7 months.

To evaluate the suspected drug trigger, patch testing was performed several weeks after resolution of the eruption according to ESCD recommendations. Commercial preparations of amoxicillin, amoxicillin/clavulanic acid, cefuroxime, clarithromycin, cetirizine, prednisolone and topical corticosteroid series were tested. Occlusion time was 24 hours according to our institutional protocol, with delayed readings performed at 24, 48, and 72 hours. Positive delayed-type hypersensitivity reactions to amoxicillin and amoxicillin/clavulanic acid confirmed a drug-induced mechanism consistent with AGEP (Fig 1, *D* and *E*).

## Discussion

The distinction between AGEP and generalized pustular psoriasis (GPP) remains challenging, as increasing evidence suggests substantial clinical, genetic, and immunopathological overlap between these entities.^2^ In our patient, the acute onset following antibiotic exposure, flexural predominance, absence of prior pustular psoriasis, compatible histopathology, and positive patch testing favored AGEP as the predominant diagnosis despite a history of guttate psoriasis.

AGEP is generally considered to be a self-limiting condition after discontinuation of the causative medication, which usually resolves within 1 to 2 weeks. In our patient, however, the disease progressed over 2 weeks despite discontinuation of antibiotics and systemic corticosteroids. Although spontaneous remission cannot be completely ruled out in a single case report, the abrupt and almost complete improvement within 1 week of starting treatment with bimekizumab suggests a relevant therapeutic effect rather than a natural course of the disease alone.

AGEP pathogenesis involves activation of drug-specific CD4+ and CD8+ T cells and release of proinflammatory cytokines such as IL-8, IL-36, IL-17, GM-CSF, and TNF-α, ultimately driving massive neutrophil recruitment.^1^^,^^3^

Recent studies highlight the relevance of the IL-17 axis in neutrophilic dermatoses, including AGEP. Both IL-17A and IL-17F are upregulated in lesional skin and produced by Th17 cells and innate immune populations.^4^^,^^5^

Selective IL-17A inhibitors such as secukinumab and ixekizumab have shown success in several AGEP case reports,^6^^,^^7^ underscoring the role of IL-17A. However, these agents do not address whether additional IL-17F blockade may provide incremental benefit.

Our case adds new perspectives to this discussion. Treatment with bimekizumab—a dual IL-17A/IL-17F inhibitor—resulted in an exceptionally rapid response, with near-complete resolution within 1 week. Such a rapid improvement supports the concept that IL-17F may play a complementary and clinically relevant role in the pathogenesis of AGEP. Additional support for this hypothesis comes from recent findings demonstrating overlaps in the genetic and immunological pathways of AGEP and generalized pustular psoriasis (GPP), including dysregulation of the IL-36 signaling pathway.^8^ The efficacy of bimekizumab in both conditions reinforces the assumption of a shared IL-17A/IL-17F-driven pustular inflammatory signature.

## Conclusion

This case demonstrates rapid, complete, and sustained resolution of severe steroid-refractory AGEP with bimekizumab, suggesting that dual IL-17A/IL-17F inhibition may represent a promising therapeutic option. Further mechanistic studies and clinical experience are needed to clarify the relative contributions of IL-17A versus IL-17F and to determine whether dual inhibition offers advantages over IL-17A-selective agents.

## Conflicts of interest

None disclosed.
